# Pathophysiology of NAFLD and NASH in Experimental Models: The Role of Food Intake Regulating Peptides

**DOI:** 10.3389/fendo.2020.597583

**Published:** 2020-11-26

**Authors:** L. Kořínková, V. Pražienková, L. Černá, A. Karnošová, B. Železná, J. Kuneš, Lenka Maletínská

**Affiliations:** ^1^Institute of Organic Chemistry and Biochemistry, Czech Academy of Sciences, Prague, Czechia; ^2^Institute of Physiology, Czech Academy of Sciences, Prague, Czechia

**Keywords:** peptides, leptin, glucagon-like peptide-1, ghrelin, non-alcoholic steatohepatitis

## Abstract

Obesity, diabetes, insulin resistance, sedentary lifestyle, and Western diet are the key factors underlying non-alcoholic fatty liver disease (NAFLD), one of the most common liver diseases in developed countries. In many cases, NAFLD further progresses to non-alcoholic steatohepatitis (NASH), fibrosis, cirrhosis, and to hepatocellular carcinoma. The hepatic lipotoxicity and non-liver factors, such as adipose tissue inflammation and gastrointestinal imbalances were linked to evolution of NAFLD. Nowadays, the degree of adipose tissue inflammation was shown to directly correlate with the severity of NAFLD. Consumption of higher caloric intake is increasingly emerging as a fuel of metabolic inflammation not only in obesity-related disorders but also NAFLD. However, multiple causes of NAFLD are the reason why the mechanisms of NAFLD progression to NASH are still not well understood. In this review, we explore the role of food intake regulating peptides in NAFLD and NASH mouse models. Leptin, an anorexigenic peptide, is involved in hepatic metabolism, and has an effect on NAFLD experimental models. Glucagon-like peptide-1 (GLP-1), another anorexigenic peptide, and GLP-1 receptor agonists (GLP-1R), represent potential therapeutic agents to prevent NAFLD progression to NASH. On the other hand, the deletion of ghrelin, an orexigenic peptide, prevents age-associated hepatic steatosis in mice. Because of the increasing incidence of NAFLD and NASH worldwide, the selection of appropriate animal models is important to clarify aspects of pathogenesis and progression in this field.

## Introduction

**Figure 1:**
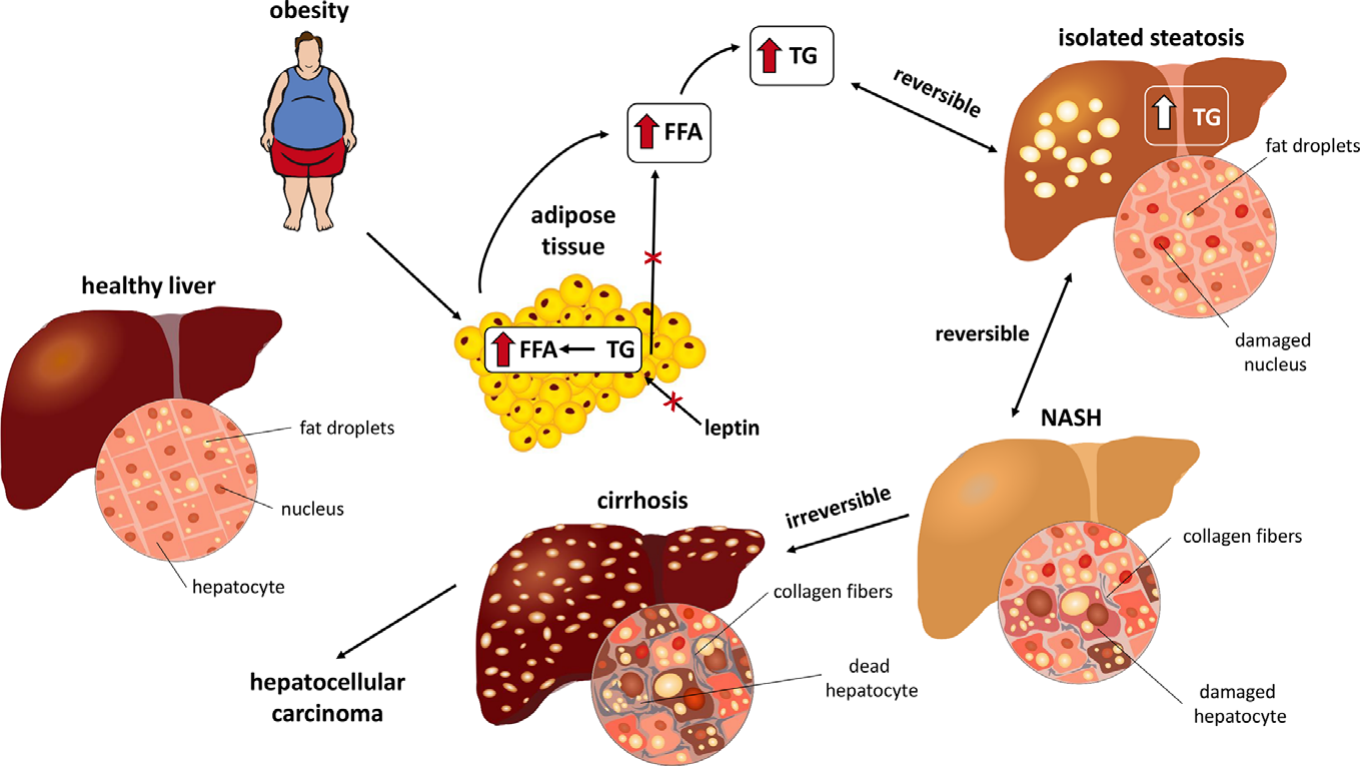
Stages of NASH.

Chronic liver diseases represent a major global health problem (1). In addition to genetic factors, various other stimuli, such as diet, metabolic diseases, etc. can alter liver function, especially the intracellular accumulation of lipids in hepatocytes. If these stimuli act for a sufficient time period, steatosis can induce inflammation resulting in non-alcoholic steatohepatitis (NASH). NASH, an extremely advanced form of non-alcoholic fatty liver disease (NAFLD), is defined as hepatic steatosis with inflammation and hepatocyte injury. NASH can eventually lead to advanced fibrosis, liver cirrhosis and liver failure (**Figure 1**). Over the past 20 years, the incidence of NAFLD has more than doubled, and it is now one of the most common liver diseases in Western countries (1). In the United States (US), the rates of prevalence of hepatic steatosis and NAFLD were estimated to 24.13%; however, it can vary by the ethnicity. It is reported that the highest prevalence is in the Hispanic Americans, followed by Americans of Europe descent and then African Americans (2). Moreover, only 3-5% of biopsy-proven NASH in the US population has been convincingly shown to progress to cirrhosis, liver failure and hepatocellular carcinoma (3) and NASH-associated cirrhosis is currently the third most frequent reason for liver transplantation. The epidemiological data from individual states of Europe suggested that approximately one-quarter of the European population is affected by NAFLD (2). As expected, the prevalence of NAFLD is substantially increased with type 2 diabetes mellitus (T2DM) and with increased body mass index, as demonstrated across Europe (2).

The pathogenesis of NAFLD and NASH appears to be multifactorial and many mechanisms have been proposed as possible causes of fatty liver infiltration (4). The association of steatosis with a number of different clinical conditions has been suggested. Common metabolic diseases such as central obesity, T2DM and hyperlipidemia are well-established risk factors and have been associated with both benign liver steatosis and progressive NASH (**Figure 1**). Moreover, it has recently been proposed that metabolic syndrome may play a causal role in the pathogenesis of NASH. Management of both NAFLD and NASH has recently become a major challenge to healthcare systems, and many different interventions have been proposed (5). In this case, one may speculate that the treatment of obesity might reduce or stop the development of NASH. The treatment of obesity is mainly related to weight reduction and improvement of eating habits. All patients with NAFLD, whether obese or normal weight, should be informed that a healthy diet has many benefits in addition to weight reduction. They should reduce the added sugar to a minimum and also minimize unhealthy fast eating and, conversely, increase fiber intake. An increase in physical activity should also be recommended. It is likely that there will not be only one right approach for all patients with NAFLD, it will be necessary to adapt the diet individually, including the inclusion of n-3 fatty acids, foods with higher monounsaturated fatty acids, fruits, vegetables and reducing the intake of saturated fats or simple carbohydrates (6, 7). Recently, it was proposed that food intake regulating peptides play a significant role in obesity regulation and may have the potential to be a drug for obesity treatment (8–11). Nevertheless, to elucidate the detailed pathophysiological mechanisms of NAFLD and NASH, appropriate experimental models need to be used. Because there are many causes of human steatohepatitis pathology, it is difficult to establish a universal experimental model. Thus, several genetic, nutritional (diet-induced) and other mouse, rat, and rabbit models have been established (12–15). Models based on overnutrition with adipose tissue enlargement and resulting metabolic complications, particularly insulin resistance, may be most useful to investigate critical etiopathogenic factors. Not only environmental factors, but also genetics play a role in the development and progression of NAFLD, as reviewed by (16). At least four genetic variants that play a role in lipid metabolism in the liver are robustly associated with the development and progression of NAFLD in humans. Patatin-like phospholipase domain-containing protein 3 (*PNPLA3*), involved in lipid droplet remodeling, is the most robust and replicable genetic variant associated with NAFLD. Transmembrane 6 superfamily member 2 (*TM6SF2*) is involved in very low-density lipoprotein (VLDL) secretion. Other genes causing the development of NASH are membrane bound O-acyltransferase domain-containing 7 (*MBOAT7*) and glucokinase regulator (*GCKR*). Additionally, other genetic variants involved in the regulation of lipid metabolism, inflammation, insulin signaling, oxidative stress and fibrogenesis in NAFLD progression have been studied (16).

This review explores the role of food intake regulating peptides in the pathology of NAFLD and NASH. Special attention will be paid to several anorexigenic peptides, such as leptin, glucagon-like peptide-1 (GLP-1), and to the orexigenic peptide ghrelin. Because anorexigenic peptides are currently promising substances in the treatment of obesity, they may also play an important role in the future treatment of liver pathology. On the other hand, the inhibition of the orexigenic peptide ghrelin could prevent age-associated hepatic steatosis. Another aim of our review is the critical view of experimental models for the study of NAFLD and NASH.

## Features of NASH

The individual markers for NASH, such as biochemical markers and inflammatory or fibrogenic factors, improve our understanding of disease pathogenesis and allow therapies to be developed. Biochemical markers measured in plasma or the liver, such as alanine aminotransferase (ALT) and aspartate aminotransferase (AST), reflect nonspecific hepatocellular damage. Aminotransferase levels can be increased two to four times the upper normal limit in NASH. Other biochemical markers such as triglyceride (TG) and cholesterol levels are measured in plasma and from liver biopsies. A high content of liver hydroxyproline (originating mostly from collagen) was also observed in obese leptin‐deficient (*ob/ob*) mice, in both obesity and NASH mouse models (17). On the other hand, inflammatory cytokines and chemokines such as tumor necrosis factor α (TNFα), interleukin (IL)-6, chemokine CC ligand-2 (CCL2) and another inflammation marker, C-reactive protein (CRP), are among the main markers of NASH (18–20). Moreover, liver biopsy is used to verify or diagnose the stage of NASH and to monitor histopathological changes in NASH. To monitor these histopathological changes, several histological scoring systems, including the NAFLD activity score (NAS) and the fibrosis scoring system, were established. The NAS system evaluates the severity of macrovesicular and microvesicular steatosis, hepatocellular ballooning or lobular inflammation. Fibrosis is scored in 7 grades with the Laennec scoring system, in which 0 indicates no fibrosis; 1, minimal fibrosis; 2, mild fibrosis; 3, moderate fibrosis; 4A, cirrhosis, mild, definite, or probable; 4B, moderate cirrhosis; and 4C, severe cirrhosis (21). However, noninvasive approaches such as magnetic resonance spectroscopy provide a sensitive method to detect hepatic steatosis (22). Other inflammatory and fibrotic markers discovered by liver mRNA sequencing include the inflammatory marker cluster of differentiation 68 *(CD68)*, which is highly expressed in macrophages, the cluster of differentiation molecule 11b *(CD11b)* marker expressed in Kupffer cells (also known as stellate macrophages located in the liver) and fibrosis markers, such as collagen type 1 α 1 chain *(col1a1)*, collagen type 3 α 1 chain *(col3a1)* and α-smooth muscle actin *(α-SMA)* (17, 21, 23, 24).

## Mouse Models of NASH

Various mouse models that mirror both the pathophysiology and the histopathology of NAFLD/NASH have been developed to elucidate the progression of NAFLD to NASH and its link to metabolic syndrome. Dietary approaches including a high-fat diet (HFD) and atherogenic and methionine- and choline-deficient (MCD) diets with many variations produce different severity of disease and are widely used (**Table 1**) (25). Other commonly used models are mice with genetic manipulations that allow to study different lipid pathways or glucose metabolism during the development of steatosis or fibrosis (**Table 1**). Toxin-induced models are not very commonly used; however, these models also develop some features of NAFLD and NASH with connection to metabolic syndrome (**Table 1**).

**Table 1:** Number of publications in mouse models of NASH.

Mouse models of NASH			Number of publications
Nutritional (dietary) models	Fat-enriched diets: HFD, HF/HFr, HF/Hsucrose, FFC		> 5,000
	Atherogenic diet		> 5,000
	MCD diet		418
Genetic models	Impairment of leptin function	*ob/ob* mice	> 1,000
		*db/db* mice	> 1,000
	Impairment of FA oxidation	Carnitine deficiency	66
		Deletion of *PPARs*	717
	Mutation of keratin 8 and 18		18
	Mutation of *SREBP-1c*		346
	Less common genetic models	KKAy mice	256
		Mutation of Fatty acid translocase (CD36)	96
		PTEN deficiency	436
Toxin/drug-induced models	STZ/Diabetes model		20
	MSG model		109
	DDC		13

This table represents number of publications found in Pubmed using key words “name of model and liver and mice” published between 1950 and 2020.

DDC, 3,5-diethoxy-carbonyl-1,4-dihydrocollidine; FA, fatty acid; FFC, high-fat diet with high fructose and cholesterol diet (FFC); JVS, juvenile visceral steatosis; HFD, high-fat diet; HF/HFr, high-fat diet with high-fructose; MCD, methionine- and choline-deficient; MSG, monosodium glutamate; PPAR, peroxisome proliferator-activated receptor; PTEN, phosphatase and tensin homolog; SREBP-1c, sterol regulatory element binding protein 1c; STZ, streptozotocin.

### Nutritional (Dietary) Models

#### High-Fat Diet

Dietary models, including obesogenic and nutrient-deficient models, can effectively trigger the development of NAFLD/NASH. Obesogenic models imitate overnutrition and a sedentary lifestyle leading to overweight or obesity in humans; therefore, the use of diet-induced obesity (DIO) models represents the natural development of NASH. The classic HFD contains 45%–75% of total calories from fat, without any nutrient deficiencies, and represents the most commonly used model of obesity in rodents (**Table 2**) (62). Mice fed this diet develop obesity with an increase in adiposity and metabolic syndrome (63, 64) and display severe liver steatosis with micro- and macrovesicular lipid accumulation and increased total liver TG but without marks of fibrosis (26–28). However, the length of diet administration, the content and type of fat used, the sex, species, and genetic background of the model can play roles in adiposity and subsequent NAFLD development.

**Table 2:** Mouse model and features of NAFLD/NASH.

Mouse model of NASH	BW	Metabolic profile	Liver histology	Liver markers	References
HFD	↑	↑ leptin ↑ insulin, ↑ glucose (plasma), ↑ TG (liver)	macro- and microvesicular steatosis no fibrosis		(26–28)
HF/HFr diet	↑	↑ insulin ↑ cholesterol ↑ TG (plasma), ↑ ALT (plasma)	necroinflammation		(29–32)
FFC diet	↑	↑ cholesterol (plasma) ↑ALT ↑AST (plasma), ↑TG (liver)	steatosis fibrosis inflammation	↑mRNA of col1a1 ↑mRNA of col1a2 ↑mRNA of TNFα	(33–35)
Atherogenic diet		↑ ALT ↑ AST (plasma), ↑ cholesterol (plasma)	steatosis cellular ballooning fibrosis inflammation		(36, 37)
MCD diet		↓ glucose ↓ insulin, ↓ leptin (serum), ↑ lipids (liver)	macrovesicular steatosis fibrosis inflammation	↑ mRNA of hepatic col1a1 and TGFβ1	(38, 39)
*Ob/Ob* mice on a FFC diet	↑	↑ insulin ↑ glucose (plasma), ↑ lipids (liver) ↑ cholesterol (plasma) ↑ ALT, ↑ AST (plasma) ↑ TG, ↑ TC (liver)	macrovesicular steatosis inflammation no fibrosis hepatocellular ballooning fibrosis inflammation	↑ col1a1 ↑ galectin-3	(17, 33)
*Db/Db* mice	↑	↑ glucose ↑leptin (serum),	macrovesicular steatosis no fibrosis		(40, 41)
Carnitine deficiency mice	↓	↓ glucose ↓ carnitine (serum), ↑ ammonia (serum) ↑ total lipids, ↑ TG (liver)	microvesicular steatosis no necrosis		(42)
*PPARα* deletion mice on a HFD *PPARβ/δ* deletion mice Fed CCl_4_ *PPARγ* deletion mice *PPARγ* liver deletion		↑ TG (liver) ↑ ALT, ↑ TNF ↑ FFA, TG ↑ insulin	steatosis steatosis protected against steatosis on HFD	↓ expression of proinflammatory genes ↓ expression of proinflammatory genes	(43–45)
Mutation of K8 mice Mutation of K18 mice with DDC		↑ ALT ↑ AST (serum),	necrotic foci steatosis tumors macrovesicular steatosis	MDBs	(46, 47)
*SREBP-1c* deficient mice *SREBP-1c* overexpression		↓ TG ↓ cholesterol (plasma), ↑ cholesterol (liver) ↑ FA (liver) ↑ TG ↑ FFA, ↑ insulin (serum),	steatosis		(48, 49)
KKAy mice	↑	↑ insulin ↑ glucose (plasma),	microvesicular steatosis	↑ lipogenesis in liver	(50)
CD36 deficient mice CD36 overexpression		↑ cholesterol ↑ NEFA (serum), ↑ TAG (serum) ↓ glucose (serum) ↑ VLDL (HFD)	resistant to steatosis on high-carbohydrate liquid diet ↓ steatosis o HFD		(51, 52)
PTEN	no changes	↑ TG (liver) ↑ ALT ↑ AST (serum),	microvesicular steatosis no fibrosis		(53)
STZ mice		↑ glucose (serum) ↑ ALT, ↑ AST (serum) ↑ lipids, ↑ TG (liver)	macrovesicular steatosis inflammation fibrosis carcinoma	↑ F4/80^+^ macrophages	(54–56)
MSG mice	↑	↑ glucose, ↑ insulin ↑ total cholesterol ↑ TG	steatosis cellular ballooning fibrosis inflammation carcinoma		(57–59)
DDC			fibrosis	↑ CD11b MDBs	(60, 61),

#### High-Fat Diet With High-Fructose/Sucrose

To increase hepatic fibrosis and preserve steatosis, HFD with high-fructose or high-sucrose (HF/HFr or HF/Hsucrose) consumption can be used; moreover, this approach has been linked to the development of NASH (**Table 2**) (65, 66). The metabolism of fructose differs from that of glucose. Hepatic metabolism of fructose favors *de novo* lipogenesis, and fructose stimulates the synthesis of TG and free fatty acids (FFAs). Fructose overconsumption is related to the obesity epidemic (67). In addition, high fructose consumption and increased fat intake is reported to be a risk factor for the development of NAFLD (29, 68). Fructose consumption, even in the absence of obesity, causes serious changes in the liver, such as dyslipidemia, insulin resistance, and NAFLD, with areas of inflammation (30). Compared to lean controls, male mice fed a high-fat, high-fructose (HFr) diet alone or combined with a high-fat (HF/HFr) diet had increased plasma cholesterol and TG and increased homeostasis model assessment for insulin resistance (HOMA-IR). Nevertheless, a dramatic increase in body mass was observed only in the HFD and HF/HFr groups and not in the HFr group. All three groups displayed features of NAFLD, with increased lipogenesis mediated by sterol regulatory element binding protein 1c (SREBP-1c), peroxisome proliferator-activated receptor γ (PPARγ) and reduced peroxisome proliferator-activated receptor α (PPARα). Inflammation was observed in the HFr and HF/HFr groups, leading to the development of NASH (30). It was reported that mice fed a high-fat, high-carbohydrate diet and a 55% fructose and 45% sucrose solution for 16 weeks developed obesity and a NASH-like phenotype with significant fibrosis (31). Mice fed a high-fat, high-sucrose diet developed severe hepatic steatosis with low-grade inflammation and fibrosis and increased inflammatory and fibrosis marker expression (32).

Mice fed a high fat, high-fructose and high-cholesterol (FFC) diet demonstrated increased body weight, plasma cholesterol, ALT, and AST and hepatic TG. Mice presented steatosis with an NAS score of 3 and fibrosis with a score of 1–3 and increased expression of fibrillary collagens such as col1a1 and collagen type 1 α 2 chain (col1a2) (33–35, 69). These mice also exhibited leukocyte infiltration in the liver and high expression of monocyte chemotactic protein-1 and TNFα (34).

The consumption of a HFD alone or a HFD enriched with other mentioned components greatly mimics features of human obesity and steatosis. However, only low-grade fibrosis and inflammation develop with the consumption of different types of HFDs. Therefore, the addition of fructose or cholesterol could enhance all features of NASH.

#### Atherogenic (High-Cholesterol, High-Cholate) Diet

An atherogenic diet enriched with cholesterol and cholic acid (or sodium cholate) has been widely used to study atherosclerosis and coronary heart disease (**Table 2**). A diet supplemented with 1.0% cholesterol and 0.3% sodium cholate increased body weight and led to elevated AST, ALT and cholesterol in Wistar rats. The liver displayed features of increased steatosis, hepatic necrosis, macrophage infiltration and hepatic fibrosis (36). An atherogenic diet induced dyslipidemia, lipid peroxidation and stellate cell activation leading to precirrhotic steatohepatitis after 24 weeks on the diet, and in contrast to HFD, cellular ballooning, one of the features of NASH, was observed. An atherogenic diet increased the expression of genes for fatty acid (FA) synthesis, oxidative stress, inﬂammation, and ﬁbrogenesis, which were further accelerated by the addition of a HFD but did not change body weight. This model suggests the critical role of lipids in causing oxidative stress and insulin resistance leading to steatohepatitis (37). Hypercholesterolemia should be considered a risk factor for hepatic fibrosis, and it could be enhanced to develop all metabolic features when a high content of cholesterol is combined with a high content of fat in the diet.

#### Methionine- and Choline-Deficient Diet

Nutrient-deficient models with low contents or lacking certain nutrients could also be used for the development of NAFLD/NASH features. The MCD diet contains high sucrose (40%) and fat (10%) but lacks methionine and choline, which are essential for hepatic β-oxidation and VLDL production (**Table 2**). Rats fed an MCD diet develop macrovesicular steatosis, inflammation, and hepatic fibrosis (70). In mice, the MCD diet caused liver injury, which is associated with hepatic microsomal lipid peroxidation. Livers revealed macrosteatosis and inflammation, together with perivenular and pericellular fibrosis (71). In different strains and animal models, the severity of NASH induced by an MCD diet differed; however, C57BL/6 mice developed the most inflammation and necrosis and best approximated the histological features of NASH (72). Although the MCD diet causes severe inflammation, oxidative stress, mitochondrial damage, apoptosis, and fibrogenesis, the metabolic profile of the model is the opposite of that of typical human NASH (73).

Specifically, mice fed an MCD diet show weight loss, decreased fasted glucose, no insulin resistance and low insulin and leptin levels (38, 39). On the other hand, the main advantage of the MCD diet is that it is easy to obtain and use.

### Genetic Models

#### Impairment of Leptin Function

##### Ob/ob Mice With Leptin Deficiency

*Ob/ob* mice carry a spontaneous mutation in the leptin gene (74) but do not have impaired leptin receptors. *Ob/ob* mice develop severe obesity due to hyperphagia and reduced energy expenditure (**Table 2**). Hyperinsulinemia, insulin resistance and hyperglycemia develop before obesity occurs (75, 76). Leptin-deficient mice are predisposed to develop severe steatohepatitis with macrovesicular steatosis; however, when maintained on a standard chow diet, they do not develop fibrosis (35). *Ob/ob* mice are protected from fibrosis because leptin is a mediator of hepatic fibrosis during chronic toxic liver injury (77). Nevertheless, leptin-deficient mice maintained on an FFC diet are more susceptible to developing features of NASH, such as steatohepatitis and fibrosis than wild-type C57BL/6J mice (33). Generally, it is believed that additional stimuli, such as an MCD diet or toxin exposure, must be added to develop inflammation and fibrosis, but interestingly, *ob/ob* mice fed a MCD diet develop moderate lobular inflammation and no detectable fibrosis (40, 77).

*Ob/ob* mice on an FFC diet displayed strong features of NASH with fibrosis, elevated plasma ALT, AST and total cholesterol, and high expression of *col1a1*, a marker of fibrosis, and *galectin-3*, a marker of inflammation (17).

##### Db/db Mice With Leptin Receptor Mutation

*Db/db* mice have a spontaneous mutation in the leptin receptor, and therefore, they are resistant to the effects of leptin (78, 79). These mice are hyperphagic, and early onset morbid obesity occurs with severe T2DM, insulin resistance, hyperglycemia and hyperleptinemia (**Table 2**) (80, 81). *Db/db* mice have macrovesicular hepatic steatosis, but they have only modestly increased liver TG compared to the control mice and do not spontaneously develop steatohepatitis or fibrosis. When *db/db* mice are exposed to a MCD diet, they exhibit increased inflammation and serum ALT compared with *db/db* mice fed a control diet and even *ob/ob* mice. Moreover, *db/db* mice fed a MCD diet revealed pericellular fibrosis (40). Interestingly, hepatic steatosis and insulin resistance, as well as body weight and adiposity, were reversed to the level of control mice in *db/db* mice by caloric restriction, a condition under which mice receive a restricted amount of food (2 g/day) (41).

#### Impairment of FA Oxidation

FA oxidation takes place in three cellular organelles: mitochondria, peroxisomes and the endoplasmic reticulum (ER) (microsomes). Mitochondrial β‐oxidation is the main route for the metabolism of FAs under normal physiological conditions (82). Deficiencies of the enzymes involved in FA oxidation have been recognized as important causes of the pathogenesis of macrovesicular and microvesicular hepatic steatosis (**Table 2**).

##### Carnitine Deficiency Leading to Juvenile Visceral Steatosis Mice

Juvenile visceral steatosis (JVS) mice have a systemic carnitine deficiency caused by mutation of the organic cation/carnitine transporter 2 gene, which encodes a plasma membrane carnitine transporter (**Table 2**) (42, 83). JVS mice develop severe lipid accumulation in the liver from an early age, with microvesicular swelling of hepatocytes, hypoglycemia, high levels of ammonia in serum, and growth retardation (42, 83, 84). This model can represent a model to examine changes in FA metabolism in the liver.

##### Deletion of Peroxisome Proliferator-Activated Receptors

Peroxisome proliferator-activated receptors (*PPARs*) belong to the nuclear receptor transcription factor family. This class comprises three *PPAR* isoforms, namely, α, β/δ, and γ, which are expressed in various tissues; *PPARs* play a role in the transcriptional regulation of glucose and lipid metabolism (**Table 2**) (85, 86).

*PPARα* is ubiquitous, but it is most highly expressed in the liver. It has a critical role in the regulation of FA uptake, β-oxidation, ketogenesis, bile acid synthesis, and TG turnover (85). *PPARα* was suggested to have an anti-inflammatory role in the liver and in adipose tissue. Mice lacking *PPARα* are more susceptible to the negative effects of a HFD, and feeding them a HFD results in increased steatosis and oxidative stress and inflammation markers compared to their controls (43).

*PPARβ/δ* is highly expressed in skeletal muscle, adipose tissue, and skin. *PPARβ/δ* is also expressed in the liver, hepatocytes, Kupffer cells and hepatic stellate cells, suggesting a potential role in inflammation and fibrosis (85). A study with *PPARβ/δ*-deficient mice suggested that *PPARβ/δ* is hepatoprotective against chemically induced hepatotoxicity, such as carbon tetrachloride (CCl_4_), by downregulating the expression of proinflammatory genes (87).

*PPARγ* is highly expressed in adipose tissue, where it controls adipocyte differentiation, adipogenesis, and lipid metabolism, but the expression of *PPARγ* in the liver is very low (85). Mice with adipose tissue-specific *PPARγ* deficiency have increased plasma FFA and TG, fatty liver, and enhanced hepatic gluconeogenesis (88). Moreover, these mice were significantly more susceptible to HFD-induced steatosis, hyperinsulinemia, and insulin resistance than their controls. *Ob/ob* mice with *PPARγ* deficiency in the liver had increased serum TG and FFA and decreased mRNA expression of hepatic lipogenic genes compared with their controls. A deficiency in hepatic *PPARγ* further aggravated the severity of diabetes in *ob/ob* mice due to decreased insulin sensitivity in muscle and fat. Hepatic *PPARγ* plays a critical role in the regulation of TG content and in the homeostasis of blood glucose and insulin resistance in steatotic diabetic mice (89).

Models with *PPAR* deficiencies are used to study the role of *PPARs* in glucose and lipid metabolism, but studies on their role in the development of NASH features could be considered rather uncommon.

#### Mutation of Keratin 8 and Keratin 18

Keratins belong to a large family of intermediate filaments, and they are expressed in epithelial cells as specific keratin pairs. Keratin 8 (K8) and keratin 18 (K18) are expressed mainly in adult hepatocytes, and mutations of this K8/K18 pair can lead to various liver diseases (**Table 2**) (90–92). Mice expressing mutant K8/18 represent an animal model for human chronic hepatitis and for studying the tissue-specific function of K8/18 (93). The increased frequency of keratin K8/K18 variants in NAFLD patients was previously reported (94). Keratin phosphorylation, transamidation and glycosylation (95) are involved in rearranging the keratin cytoskeleton into cytoplasmic inclusions, known as Mallory-Denk bodies (MDBs), found in specific liver diseases such as alcoholic hepatitis and cirrhosis (90).

#### Mutation of Sterol Regulatory Element Binding Protein-1c

SREBP plays a key role in lipid homeostasis by regulating the expression of genes involved in lipid synthesis (96, 97). *SREBP-1c* is transcriptionally controlled by various factors, mainly insulin and glucose, and regulates hepatic glucose and lipid metabolism (**Table 2**) (98). In cultured hepatocytes, insulin and glucose activate the transcription of the *SREBP-1c* gene, whereas glucagon has an inhibitory effect (99, 100). *SREBP-1c* expression was positively correlated with fatty acid synthase (*FASN*) expression, and higher levels were found in the liver in NAFLD models than in controls (101). The *SREBP-1* gene has a role in the genetic predisposition of metabolic diseases such as obesity, T2DM, and dyslipidemia (102).

*SREBP-1c* deficiency led to the reduced expression of enzymes involved in FA and TG synthesis. *SREBP-1c* KO mice had reduced total plasma TG and cholesterol. In contrast, the liver cholesterol content was higher in *SREBP-1c* KO mice than in WT mice (48). Mice with liver-specific overexpression of human *SREBP-1c* (alb-*SREBP-1c*) developed hepatic lipid accumulation featuring a fatty liver by the age of 24 weeks. Moreover, liver-specific over-expression of human SREBP-1c (alb-*SREBP-1c*) mice had increased liver FA levels, serum TG and FFA, and insulin levels, indicating insulin resistance (49).

#### Less Common Genetic Models

##### KKAy Mice

Yellow *KKAy* mice carry the yellow obese gene (*Ay*) and they develop spontaneous obesity due to hyperphagia with increased adiposity and diabetic symptoms. KKAy mice are characterized by insulin resistance with increased blood glucose, circulating insulin levels and increased lipogenesis in the liver and in adipose tissue (**Table 2**) (103). The livers of KKAy mice had microvesicular steatosis and an increased degree of hepatic steatosis (50). KKAy mice represent a model that closely resembles obesity and T2DM in humans, who develop several metabolic diseases therefore, this model is valuable for the development of potential therapeutic strategies not only for diabetes (104).

##### Deficiency or Overexpression of Fatty Acid Translocase (CD36)

Fatty acid translocase, or cluster of differentiation 36 (CD36), is a transmembrane glycoprotein that facilitates lipid transport (105). The circulating serum level of CD36 is increased in NAFLD and correlates with the histological grade of steatosis intrahepatic lipids, ALT and TG (106, 107). CD36-deficient mice showed a significant increase in fasting levels of cholesterol, non-esterified fatty acid (NEFA) and TG with lower fasting serum glucose levels (**Table 2**) (51). CD36 KO mice are resistant to hepatic steatosis when fed a high-carbohydrate liquid diet, and they do not develop alcoholic steatosis when chronically fed alcohol (108). CD36 was suggested to be a protective metabolic sensor in the liver under lipid overload and metabolic stress (52).

##### Mutation of Phosphatase and Tensin Homolog in the Liver

Phosphatase and tensin homolog (*PTEN*) is a tumor suppressor gene mutated in many human cancers but has multiple roles in organisms. Its expression is reduced or absent in almost half of hepatoma patients (109, 110). The hepatocyte-specific null mutation of PTEN in mice (PTEN-HEP-KO) showed marked hepatomegaly and steatohepatitis with TG accumulation, a phenotype similar to that observed in human NASH (**Table 1**). PTEN deficiency in hepatocytes led to steatosis through increased FA uptake and *de novo* lipogenesis (53).

### Toxin/Drug-Induced Models

#### Streptozotocin Diabetes Model

Streptozotocin (STZ) is a broad-spectrum antibiotic with antitumor, oncogenic, and diabetogenic properties (111). Multiple small intraperitoneal injections of STZ (40 mg/kg) in mice produce pancreatic insulitis, with the selective destruction of pancreatic β cells and diabetes mellitus (112), causing insulin deficiency, hyperglycemia, polydipsia, and polyuria, all of which mimic human type 1 diabetes mellitus (**Table 2**) (113). Hepatic changes, including lipid peroxidation, mitochondrial swelling, peroxisome proliferation and hepatocyte proliferation inhibition, occur before STZ induces hyperglycemia (114). In the subacute phase, intraperitoneal injection of STZ resulted in an increase in serum glucose and in serum and liver lipids and a decrease in liver glycogen (54). Neonatal male mice exposed to low-dose STZ developed liver steatosis with diabetes after one week on a HFD. With continuous HFD, neonatal STZ mice develop NASH pathology with decreased hepatic fat deposits and increased lobular inflammation and fibrosis. At older ages, mice develop hepatocellular carcinoma (55). Neonatal STZ mice demonstrated focal liver lesions and hepatocellular carcinoma. Hematoxylin-eosin staining showed macrovesicular steatosis, lobular inflammation, hepatocellular ballooning and mild fibroblast proliferation by silver impregnation (56). This model represents a model of NASH linked to diabetes and hepatocellular carcinoma without the accumulation of fat and the development of steatosis.

#### Monosodium Glutamate Model

Subcutaneous injections of monosodium glutamate (MSG) into newborn mice cause acute neuronal necrosis mainly in the arcuate nucleus (ARC) of the hypothalamus. MSG-treated mice display stunted growth due to impaired growth hormone production (115), marked obesity, and female sterility, but they are rather hypophagic (116). MSG mice had an 8 times higher fat-to-body mass ratio and developed hyperglycemia and hyperinsulinemia (**Table 2**) (117–119), which was more pronounced in males than in females. The obesity-related changes in the feeding behavior of the MSG-treated mice are possibly the result of missing leptin and insulin receptors in ARCs and consequent altered neuropeptide signaling (120). The injection of MSG in ICR mice leads to the development of significant inflammation, central obesity, and T2DM. Compared with control mice, MSG-ICR mice had increased concentrations of glucose, insulin, total cholesterol, and TG (57). MSG-ICR mice developed NAFLD and NASH-like pathology and had steatohepatitis and dysplastic nodular lesions within the fibrotic liver (121). A different strain, MSG-DIAR (ddY, Institute for Animal Reproduction, Japan) mice, revealed a similar pattern of T2DM and macrovesicular steatosis, lobular inflammation with neutrophils, and ballooning degeneration. At an older age, they developed cellular structures mimicking human hepatocellular carcinoma (58). The crucial metabolic window for studying pathophysiological events involved in NAFLD/NASH progression is considered at 4–6 months of age, the age at which MSG-treated mice also have peripheral insulin resistance (59). The MSG model develops features of NASH with obesity and steatosis, high TG, and insulin resistance; however, this model is not used frequently.

#### Porphyrinogen Agent as Inducer of NASH-Like Liver Lesions

Feeding with 3,5-diethoxycarbonyl-1,4-dihydrocollidine (DDC) leads to chronic xenobiotic-induced cholangiopathy in mice (**Table 2**). DDC feeding results in significantly increased number of CD11b-positive cells. Moreover, mice fed DDC have a biliary type of liver fibrosis (60). DDC triggered the formation of MDBs and with that, the expression of keratins 8/18 was elevated (61).

## Role of Anorexigenic Peptides in NASH

Anorexigenic peptides, of both hypothalamic and gut origin, play important roles in the pathology of NAFLD and NASH. The most studied peripheral gut hormones include cholecystokinin, leptin, amylin, GLP-1, oxyntomodulin, and bombesin, and the main hypothalamic anorexigenic peptides include cocaine- and amphetamine-regulated transcript peptide, α-melanocyte-stimulating hormone, corticotropin-releasing factor and prolactin- releasing peptide. This review is focused mainly on leptin and GLP-1 as they are the best known anorexigenic peptides involved in NAFLD and NASH pathogenesis (122, 123).

### Leptin

Leptin, a product of the *ob* gene, is a hormone secreted by white adipose tissue that acts as a major regulator of food intake and energy homeostasis. Because leptin receptors are found in the brain and in many peripheral tissues, leptin triggers many biological effects and has the potential to affect a wide range of diseases (124–127). Obese individuals usually have high plasma leptin concentrations, and this hyperleptinemia leads to leptin resistance (128). Mutations in the *ob* gene are rarely responsible for obesity in humans, but several animal models with *ob* gene mutations exist (73).

The binding of leptin to its receptor activates Janus kinase 2 (JAK2) and phosphorylates specific tyrosine residues of the receptor and downstream proteins, including signal transducer and activation of transcription (STAT3), Src homology region 2 (SH2)-containing protein tyrosine phosphatase 2 (SHP2), insulin receptor substrate 2 (IRS2), and phosphatidylinositol 3-kinase (PI3K), which regulate the transcription of genes involved in food intake and lipid metabolism. Leptin activates 5′ adenosine monophosphate-activated protein kinase (AMPK) and decreases acetyl-CoA carboxylase (ACC) activity in skeletal muscle while increasing mitochondrial β-oxidation. Another aspect of the metabolic activity of leptin is the inhibition of hepatic stearoyl-CoA desaturase-1 (SCD-1) activity, which regulates lipoprotein metabolism and energy consumption. On the other hand, leptin inactivates AMPK, increases ACC activity and decreases food intake in the hypothalamus (126).

#### Role of Leptin in NAFLD/NASH Progression to Fibrosis

Ten years ago, hepatic lipotoxicity and non-hepatic factors such as adipose tissue inflammation and gastrointestinal imbalance were linked to evolution of NAFLD (129). Nowadays, the degree of adipose tissue inflammation was shown to directly correlate with the severity of NAFLD. Consumption of higher caloric intake is increasingly emerging as a fuel for metabolic inflammation not only in obesity-related disorders but also NAFLD. Gut microbiome in NAFLD is gaining importance too (130). Characteristic features of NAFLD progression include imbalance in FA metabolism, cytokine dysregulation, and oxidative metabolic stress. Oxidative stress is mainly characterized by the excessive production of reactive oxygen species by three main mechanisms, namely, lipid peroxidation, cytokine induction, and Fas ligand (type II transmembrane protein belonging to the TNF family), and induces the progression from steatosis to steatohepatitis and to fibrosis (131). In NAFLD progression, oxidative stress causes the induction of purinergic receptor X7 expression at both the mRNA and protein levels in inflammatory cells, but the detailed mechanisms are not yet clear (132).

**Figure 2:**
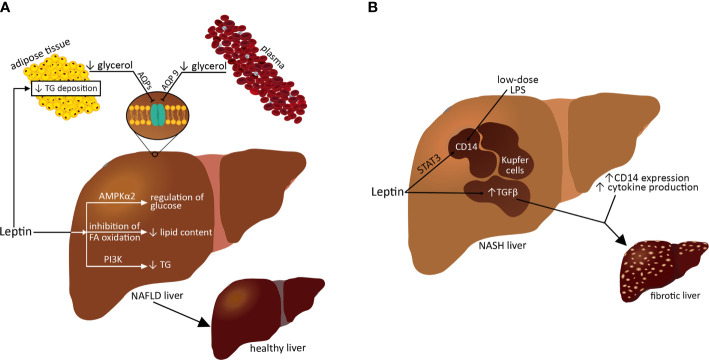
Role of leptin in NAFDL/NASH progression.

Circulating leptin levels primarily reflect energy stores in the body but also indicate acute changes in caloric intake. Leptin appears to exert a dual action in experimental NAFLD models; it protects against liver steatosis, at least in the early stages of the disease, but it also acts as an inflammatory and fibrogenic mediator when the disease persists or continues (133). An important role of leptin is to reduce the deposition of TG in adipocytes and at the same time to limit the storage of TG in nonadipose tissues, including the liver, and thus protect them from lipotoxicity and lipoapoptosis (**Figure 2**) (134). Leptin prevents liver steatosis in animal models by affecting both lipid and glucose metabolism. Under normal conditions, leptin suppresses hepatic glucose production and hepatic lipogenesis (135). Chronic central administration of leptin reduces the expression of hepatic lipogenic genes and reduces TG content by stimulating hepatic sympathetic activity; this function requires PI3K signaling because the leptin-mediated impairment of PI3K signaling leads to hepatic steatosis without inducing obesity (**Figure 2**) (133). For lipid accumulation in the liver, glycerol is needed. A decrease in glycerol availability might be involved in the beneficial effects of leptin on NAFLD. Integral membrane aquaglycerolporins (AQP3, AQP7, AQP9, and AQP10) form a channel across the cell membrane and thus facilitate glycerol transport. The main gateway facilitating the release of glycerol from adipocytes is AQP7; however, AQP3, AQP9, and AQP10 also aid in glycerol efflux from fat depots (**Figure 2**). Subsequently, circulating plasma glycerol is transferred to hepatocytes by hepatic-specific AQP9, where glycerol kinase catalyzes the initial step for its conversion to glucose (gluconeogenesis) or to TG in *ob/ob* mice (136). More specifically, leptin inhibits hepatic *de novo* lipogenesis while stimulating FA oxidation, thereby reducing lipid content in isolated livers (133, 137, 138). The steatosis observed in *ob/ob* mice suggests that leptin is taken up by the fatty liver, indirectly owing to central nerve pathways and directly by hepatic AMPK activation. However, the regulation of glucose production in the liver by leptin but not insulin requires hepatic AMPKα2 activity (131). On the other hand, leptin may promote hepatic fibrogenesis through the upregulation of transforming growth factor β (TGF-β) in Kupffer cells and sinusoidal endothelial cells. Leptin prevents the upregulation of *col1a1* mRNA, a change associated with the fibrotic process in the liver. Fibrosis develops in the absence of leptin in *ob/ob* mice; therefore, liver fibrosis depends on the presence of leptin in chronic liver damage (77, 139). In Kupffer cells, leptin induces cluster of differentiation 14 (CD14) expression by activating STAT3 (140). This results in a hepatic hypersensitive response and progression from simple steatosis or liver inflammation to fibrosis (**Figure 2**). This points to possible therapeutic approaches for NASH by targeting leptin-dependent STAT3 and CD14 signaling. During liver fibrosis, the role of leptin and its functioning receptors, in particular in activated hematopoietic stem cells (HSCs), has been demonstrated. Increases in leptin-associated *col1a2* gene expression and in leptin-enhanced *col1a2* gene promoter activity were observed by ribonuclease protection analysis *in vitro* (141). Furthermore, leptin enhances platelet-derived growth factor-dependent proliferative responses in HSCs, most likely through actions involving the PI3K/Akt pathway (140, 141).

In conclusion, it seems that in the initial stages of the disease, leptin may protect against hepatic steatosis. However, when the disease progresses, leptin may act as an inflammatory and fibrogenic agent. Leptin deficiency can lead to hepatic steatosis, and excess leptin can promote hepatitis and fibrosis.

### GLP-1

**Figure 3:**
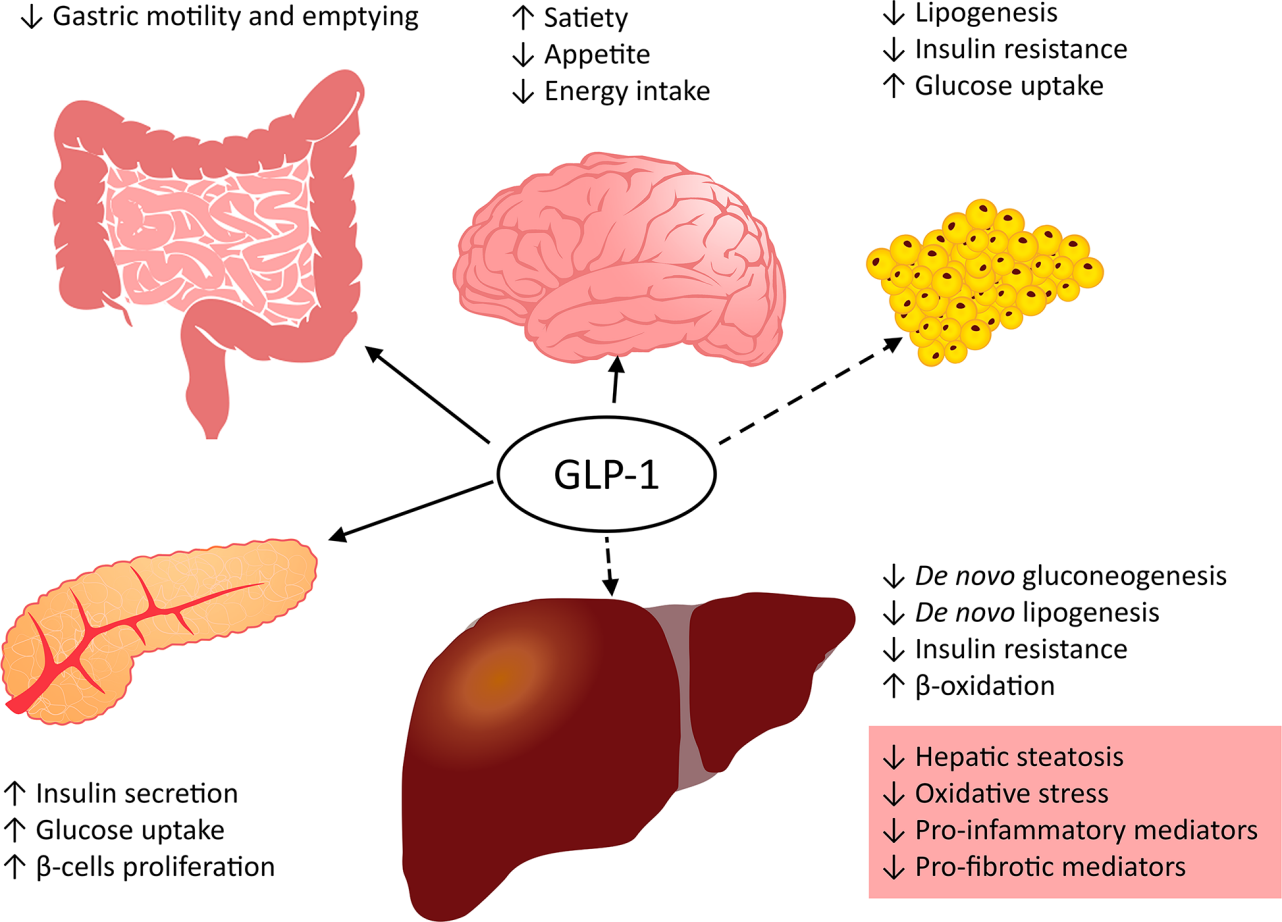
Role of GLP-1 in metabolism.

The gut incretin hormones glucose-dependent insulinotropic peptide (GIP) and GLP-1 are secreted after the oral administration of nutrients and stimulate insulin secretion, which leads to a decrease in glucose concentration (142, 143). GLP-1 slows gastric motility, enhances satiety, reduces appetite and energy intake, and suppresses postprandial glucagon secretion (**Figure 3**) (144, 145). Glucagon-like peptide-1 receptor (GLP-1R; also termed secretin-like receptor) belongs to the G protein-coupled receptor family B. GLP-1R mRNA is found in the pancreatic islets, lungs, hypothalamus, hippocampus, cerebral cortex, brainstem, kidney, stomach, intestine, skin, and heart of rodents and humans (146, 147).

#### GLP-1–Activated Pathways in Hepatic Lipid Metabolism

GLP-1R agonists stimulate the production of cAMP through adenylate cyclase, and cAMP activates protein kinase A (PKA) and exchange protein activated by cAMP (Epac) (148, 149). PKA and Epac induce insulin gene transcription and secretion (150). Activation of cAMP in turn activates PKA, which leads to the phosphorylation of AMPK (151). In addition, cAMP activation stimulates the epidermal growth factor receptor, pointing to the activation of the PI3K/Akt signaling pathway. These actions suppress the expression of genes that have a key role in the stimulation of insulin secretion affects the repression of hepatic gluconeogenesis and lipogenesis and reduce postprandial plasma glucose levels by elevating insulin-regulated glucose uptake (**Figure 3**) (148, 149). Insulin or ER stimulation is required for release *SREBP-1c*, which activates the expression of the lipogenic genes *ACC* and fatty acid synthase (*FASN*) and enhances glycolytic flux. The expression of lipogenic genes requires the activation of the transcription factors *SREBP-1c* and carbohydrate response element binding protein *(ChREBP)*. GLP-1R agonists reduce the expression of both *ChREBP* and *SREBP-1c* genes and inhibit *de novo* lipogenesis in the liver (98, 152).

*PPAR* transcription factors are also involved in the regulation of lipid metabolism. GLP-1 analogs may have a key role in increasing the expression of *PPARα* and suppressing *PPARγ* genes (153, 154).

#### The Role of GLP-1 in NAFLD/NASH Progression to Fibrosis

GLP-1R agonists are approved for the treatment of T2DM, and they have great potential in the treatment of NAFLD and NASH. GLP-1R agonists enhance insulin secretion and improve glucose tolerance, which leads to a decrease in lipogenesis *de novo* and enhances hepatic FA oxidation and lipid export (**Figure 3**). Moreover, receptor activation leads to the central regulation of satiety and decreases in appetite and energy intake. GLP-1R agonists alleviate metabolic inflammation and NASH by suppressing the expression of inflammatory genes such as TNFα, IL-6, and nuclear factor NF-kappa-B (NFκB) (155, 156). Many studies in mice show improvement and the possibility of preventing hepatic steatosis using different GLP-1R agonists (35, 157–161). GLP-1R agonists directly affect Kupffer cell function and reduce the influx of macrophages into the liver (162). Together with dipeptidyl-peptidase 4 (DPP4) inhibitors, they decrease not only proinflammatory but also profibrotic mediators. Decreasing profibrotic mediators in mice occurs *via* the reduction of *TGF-β* gene expression and other profibrotic mediators through the stimulation of cAMP production (151, 159, 163). Epac and PKA activation by cAMP leads to a reduction in col1, col3, and angiotensin II expression and to smad (TGF-β superfamily member) pathway inhibition (164, 165).

#### GLP-1R Agonists in Experimental Models

Despite the fact that food intake-regulating peptides have great potential in the treatment of metabolic diseases and NAFLD/NASH, there is very little known about their effects on current mouse models of NASH. Only GLP-1R agonists, primarily used as antidiabetic/antiobesity drugs, are used as potential agents for NAFLD/NASH treatment, as demonstrated in several studies with mouse models. The GLP-1R agonists exenatide, liraglutide and semaglutide are already used in clinical practice for the treatment of T2DM and obesity. The available mouse models of NASH address different aspects of the disease, leading to various clinical utilities in drug discovery. The advantages and limitations of current *in vivo* mouse models of NASH in view of different targets for NASH treatment are summarized in the review by Hansen et al. (23). The GLP-1R agonist Exendin-4 was originally isolated from the saliva of the Gila monster. Because of the change in the amino sequence at position 8, Exendin-4 has a longer half-life and more stable interaction with DPP4 than GLP-1 (148, 157). Treatment with Exendin-4 (synthetic form is called exenatide) reduced serum glucose levels and body weight and improved serum ALT in *ob/ob* mice. Exendin-4 also has a positive effect on hepatocyte lipid metabolism. Treatment significantly decreased hepatic lipid content and thiobarbituric acid reactive substance concentration, which is an important parameter of hepatic oxidative stress in NASH and NAFLD. Moreover, Exedin-4 significantly reduced the mRNA levels of *SREBP-1c* and *SCD-1*, parameters regulating *de novo* lipogenesis in the liver, and increased the level of *PPARα* mRNA in *ob/ob* mice (157). The results from the Trevaskis et al. study in *ob/ob* and C57BL/6 mice on a HFD support the therapeutic potential of the exendin-4 analog AC3174 in NASH and NAFLD treatment. The analog AC3174 diminished plasma TG and ALT levels and lipid accumulation in the liver and attenuated fibrosis (35). In *db/db* mice fed an MCD diet, exendin-4 treatment attenuated hepatic steatosis, TG and FFA content, oxidative stress and hepatic inflammation (166).

Several studies in mice have shown that treatment with liraglutide, a long-acting palmitoylated analog of GLP-1, alleviates hepatic steatosis and has great potential to inhibit NASH or NAFLD (160, 161, 167, 168). In two different NASH mouse obesity models, an atherogenic diet model and *ob/ob* mice, chronic treatment with liraglutide reduced body weight, lowered steatosis scores and inhibited fibrosis (through a decreased col1a1) (17). In male C57BL/6J mice fed a western diet, liraglutide significantly improved insulin sensitivity and prevented NASH pathology. Liraglutide improved lipid flux between liver and adipose tissue, downregulated genes regulating *de novo* lipogenesis and increased the expression of genes associated with β-oxidation, FA uptake and VLDL transport (167). The administration of liraglutide in male C57BL/6 mice fed a HFD reduced ALT and AST serum levels and inhibited NOD-like receptor family pyrin-containing 3 inflammasome gene expression, which has a critical role in NAFLD pathogenesis (161). Another study using (C/EBP) homologous protein (CHOP) C57BL/6 KO mice fed an FFC diet significantly attenuated hepatic steatosis after 4 weeks of liraglutide treatment in WT mice and showed that CHOPs play an important role in the protection of hepatocytes from diet-induced ER stress (160).

GLP-1R and glucagon receptor (GLP-1R/GR) dual agonists also have potential in the treatment of NAFLD. DIO C57BL/6 mice treated with GLP-1R/GR dual agonists showed improved glucose metabolism and reduced food intake and weight loss. In the liver, decreases in acetyl-CoA and malonyl-CoA were observed, and ketogenesis was increased (169). Antiobesity effects were not the only effects observed in C57BL/6J mice fed a HFD treated with GLP-1R/GR dual agonists. This study showed reduced mRNA expression of hepatic *SREBP-1c* and *SCD-1* and increased *PPARα* expression. Reduced levels of the inflammation factors TNFα and IL-6 in plasma and of TGF-ß, monocyte chemoattractant protein-1, matrix metalloproteinase-9 and TNFα in the liver inhibited the development of NASH and NAFLD (159). Moreover, a GLP-1R and GIP receptor dual agonist attenuated NASH in C57BL/6J mice fed an atherogenic diet, significantly decreased body and liver weight, decreased liver TG and improved NAS, showing synergistic action compared to monotherapy with GLP-1R agonist or GIP (170).

## GHRELIN

Orexigenic peptides such as ghrelin, neuropeptide Y, agouti-related protein, and orexins play an important role in the mechanism of food intake (171). Ghrelin peptide is released from the stomach as the endogenous ligand for the growth hormone secretagogue receptor (GHSR), which stimulates growth hormone (GH) release from the anterior pituitary gland. Ghrelin regulates food intake, adiposity, body weight, glucose metabolism, taste sensation, sleep modulation, brown fat thermogenesis, stress and anxiety responses, muscle atrophy, gut motility, gastric acid secretion, and cardiovascular function (172–174).

Ghrelin is the only known peptide with an attached FA. The octanoylation of ghrelin is catalyzed by ghrelin-O-acyltransferase. This modification is essential for the biological activity of ghrelin (175). Ghrelin without the acyl group (des-acyl ghrelin) is biologically inactive (101, 176). Des-acyl ghrelin is also present at significant levels in both blood and stomach, but des-acyl ghrelin can neither bind to GHSR nor exhibit GH release (177).

### Ghrelin-Activated Pathways in Hepatic Lipid Metabolism

Ghrelin plays a complex role in hepatic metabolism and liver diseases. On the one hand, ghrelin induces adiposity in the liver; on the other hand, ghrelin elicits a protective effect against inflammation and fibrosis. Currently, there is no clear explanation for opposite actions of ghrelin in these pathologies (178).

Central ghrelin administration leads to lipid storage in the liver, which is independent of GH. Ghrelin promotes energy storage to minimize negative effects in periods of food shortage (179).

Ghrelin has a direct peripheral effect on lipogenesis in hepatocytes. Ghrelin activates GHSR in hepatocytes, which leads to increased TG synthesis, by increasing the expression of lipogenesis-related genes in hepatocytes. These effects are mediated by mammalian target of rapamycin (mTOR) – *PPARγ* signaling pathway activation (180). The activation of this pathway is independent of the central stimulation of energy intake in the hypothalamus, where activated AMPK mediates the orexigenic action of ghrelin. Ghrelin stimulates the activity of AMPK in the hypothalamus but inhibits AMPK activity in the liver and adipose tissue, resulting in increased lipogenesis (181).

Other studies have shown that the tumor protein p53 is crucial for the stimulation of lipid storage in fat and the liver by ghrelin. Lack of p53 abolishes the stimulation of lipid storage induced by administered ghrelin (182).

In the gastrointestinal tract, ghrelin has potent anti-inflammatory properties. Exogenous ghrelin pretreatment augmented the release of the anti-inflammatory cytokine interleukin IL-10 (183) and inhibited the production of various pro-inflammatory cytokines, such as IL-1*β*, IL-6, IL-8, and TNFα (184). Furthermore, the hepatoprotective effect of ghrelin is caused by inhibition of apoptosis and proliferation stimulation in various cell types (185, 186).

### The Role of Ghrelin in NAFLD/NASH Progression to Fibrosis

The activation of the gastric ghrelin-brain axis is essential to maintain biological homeostasis. The liver damage signal caused by FA infiltration is sent to the brain and stomach *via* autonomic nerve connections, which causes an increase in ghrelin release. These signals could slow down the progression of NAFLD. Impairment of this appetite control is necessary for NASH pathology (187).

Low levels of acylated ghrelin in plasma are found in NASH (188, 189). Moreover, decreased plasma ghrelin correlates with increased immunoglobulin production that is often observed in patients with chronic liver disease (190) and correlates with liver inflammation (191). The most likely reason for the low ghrelin levels in NAFLD patients is insulin resistance. Low ghrelin levels are observed in several diseases characterized by insulin resistance, including severe obesity (192), acromegaly (193), hypogonadism (194), and polycystic ovary syndrome (195). Nevertheless, the molecular mechanisms remain elusive (196).

Patients with NASH had a twofold higher concentration of des-acyl ghrelin compared with healthy humans. Des-acyl ghrelin also correlated with ALT, AST, TG levels, fasting glucose, MDBs, and portal fibrosis, which are strongly associated with the occurrence of NASH (197).

Oxidative stress and inflammation are key factors in the development of NAFLD/NASH. Thus, the impairment of these processes could revert the development of NASH. The administration of ghrelin during and after NAFLD development reduced inflammation, apoptosis, and oxidative stress and improved lipid metabolism in the rat liver (198). In addition to the protective effects of ghrelin mentioned above, ghrelin also exerts antifibrotic and hepatoprotective effects in the injured livers of rodents (199). Antifibrotic effects can also be seen in other tissues, such as the heart (200) and colon (201).

Exogenous and endogenous ghrelin regulates fibrogenesis in mice and humans (199). Antifibrotic effects are caused by several mechanisms. Ghrelin protects hepatocytes from cell death by reducing inflammatory cells, decreasing apoptosis, and increasing the activation of hepatoprotective signaling pathways such as Akt phosphorylation. Ghrelin modulates inflammation by downregulating the NFκB pathway (202). The wound-healing response to injury is caused by decreasing oxidative stress in livers (199). Ghrelin also reduces profibrogenic cytokine TGF-β1 and p-Smad3 expression levels that are involved in increased deposition of fibronectin, col1 and α-SMA in liver fibrosis. Ghrelin suppresses autophagy, thus reducing available energy from intracellular lipid degradation (202).

## Pharmacological Therapies for NAFLD/NASH

NAFLD is associated with obesity, T2DM, dyslipidemia, and metabolic syndrome. Weight loss through dietary changes and lifestyle modifications is now the only proven effective therapy for patients with NAFLD/NASH. Nevertheless, these approaches are not sufficient for the treatment of fibrosis and even cirrhosis. Pharmaceutical companies are developing new drugs for the treatment of NASH, but no drugs have yet been approved (203, 204). The pathophysiology of NAFLD is very complex and associated with different features, such as lipotoxicity, inflammatory cytokines, apoptosis, and insulin resistance. Therefore, drugs to treat NASH could target these features (203).

In patients with T2DM, the prevalence of NAFLD is 75% (205); therefore, the use of antidiabetic drugs to improve insulin resistance could be one approach for treatment. Metformin is an insulin sensitizer used as a major therapy for T2DM because of its low cost, body weight-lowering effect and safety profile (203). Nevertheless, metformin was reported to not significantly improve liver histology and therefore is not recommended in the treatment of NASH (206).

Pioglitazone belongs to the thiazolidinedione family, agonists of *PPARγ*, and improves glucose and lipid metabolism. Treatment with pioglitazone improved insulin sensitivity, plasma ALT and AST levels and liver steatosis, inﬂammation, and ballooning (206, 207). Pioglitazone causes some side effects, such as body weight gain, possible bladder cancer and bone loss in women (206). Nevertheless, risks and benefits should be considered, as pioglitazone improves liver histology in patients with and without T2DM with NASH.

GLP-1R agonists and DPP4 inhibitors were also investigated as possible therapeutics for NASH treatment. Liraglutide, a stable GLP-1 analog, improved steatosis and hepatocyte ballooning and slowed fibrosis progression (205, 208). On the other hand, DPP4 inhibitors delay the quick inactivation of GLP-1 in plasma. Sitagliptin was unable to improve the fibrosis score or NAS or to reduce liver fat after 24 weeks of therapy (209, 210).

## Conclusions

A large number of mouse models could be used to study NASH pathogenesis and its possible treatment. However, some approaches do not coincide with human NALFD. Currently, NASH is associated with the development of prediabetes and metabolic syndrome with elevated ALT, AST, cholesterol, and FFA levels.

Genetic models represent advantages in the time required and development of concrete metabolic features associated with NAFLD. However, these mutations are very rare in humans. Moreover, some of these models fail to induce the metabolic comorbidities typically observed in humans with NASH, such as insulin resistance, obesity and dyslipidemia.

Regarding nutritional models, a large number of different approaches, with variable fat content, glucose/fructose enrichment or other substance additions or deficiencies, create complicated decisions for researchers. The crucial advantage of nutritional models is the ability to mimic human NAFLD, both pathophysiologically and phenotypically. In contrast, a longer period is necessary for the development of NAFLD, and a lesser degree of pathology is observed. Nevertheless, this issue could be overcome by the use of high glucose/fructose levels or increased cholesterol levels in the diet.

Finally, regarding chemically induced models, toxic agents are not pathophysiologically related to NASH disease. However, those models could be used to enhance fibrosis and cirrhosis leading to liver failure.

All these models should be used with caution, and their use should be limited to clearly defining liver-specific research and to studying the pathologic features of human NAFLD/NASH.

It seems that anorexigenic and orexigenic peptides are involved in the pathology of NAFLD and NASH. Leptin may have a potential dual action in NAFLD and NASH. Leptin may protect the liver from hepatic steatosis at the initial stage of the disease but also acts as an inflammatory and fibrogenic marker when the disease progresses. Leptin deficiency can lead to hepatic steatosis, and excess leptin can promote hepatitis and fibrosis. The efficacy of NASH treatment with anorexigenic leptin is questionable, similar to potential treatment with orexigenic ghrelin. Ghrelin induces adiposity in the liver, but also ghrelin elicits a protective effect against inflammation and fibrosis.

Pharmaceutical companies are developing new drugs for the treatment of NAFLD and NASH; however, no drugs have been approved yet. Because NAFLD is associated with obesity, T2DM, dyslipidemia, and metabolic syndrome, weight loss through dietary changes and lifestyle modifications is now the only proven effective therapy for patients with NAFLD/NASH. Nevertheless, these approaches are not sufficient for the treatment of fibrosis and even cirrhosis.

Recently, GLP-1R agonists used primarily as antidiabetic or antiobesity drugs have shown the greatest potential in the possible treatment of NASH. GLP-1R agonists enhance insulin secretion and improve glucose tolerance, which leads to a decrease in lipogenesis *de novo* and enhances hepatic FA oxidation and lipid export. GLP-1R agonists alleviate metabolic inflammation and NASH by suppressing the expression of inflammatory genes. Nevertheless, more attention should be paid to the potential role of other anorexigenic and/or orexigenic peptides in the pathophysiology of NAFLD and NASH in the future, especially in relation to the treatment of obesity and T2DM.

## Author Contributions

LK and LM coordinated writting of the manuscript, LK, JK, VP, LM, AK, and LC wrote the manuscript. BZ, JK, and LM revised and completed the manuscript. All authors contributed to the article and approved the submitted version.

## Funding

This work was supported by the Grant Agency of the Czech Republic (Grant No. 18-10591S).

## Conflict of Interest

The authors declare that the research was conducted in the absence of any commercial or financial relationships that could be construed as a potential conflict of interest.

## Abbreviations

ACC, acetyl-CoA carboxylase; Alb-SREBP-1c, liver-specific over-expression of human SREBP-1c; ALT, alanine aminotransferase; AMPK, 5’ adenosine monophosphate-activated protein kinase; AQPs, aquaglyceroporins; ARC, nucleus arcuatus; AST, aspartate aminotransferase; Ay, yellow obese gene; CC, chemokine; CCL, chemokine ligand-2; CCl_4_, carbon tetrachloride; CD11b, cluster of differentiation 11B; CD14, cluster of differentiation 14; CD36, cluster of differentiation 36; CD68, cluster of differentiation 68; CHOP, C/EBP homologous protein; ChREBP, carbohydrate response element binding protein; Col1a1, collagen type 1 α 1 chain; Col1a2, collagen type 1 α 2 chain; Col3a1, collagen type 3 α 1 chain; CRP, C-reactive protein; DDC, 3,5-dietoxy-carbonyl-1,4-dihydrocollidine; DIAR, ddY, Institute for Animal Reproduction; DIO, diet-induced obesity; DPP4, dipeptidyl peptidase-4; Epac, exchange protein activated by cAMP; ER, endoplasmatic reticulum; FA, fatty acid; FASN, fatty acid synthase; FFA, free fatty acid; FFC, high fructose and cholesterol; GCKR, glucokinase regulator; GH, growth hormone; GHSR, growth hormone secretagogue receptor; GIP, glucose-dependent insulinotropic peptide; GLP-1, glucagon-like peptide 1; GLP-1R, glucagon-like peptide 1 receptor; GLP-1R/GR, GLP-1R and glucagon receptor dual agonists; HF/HFr, high-fat high-fructose; HFD, high-fat diet; HFr, high-fructose; HOMA-IR, homeostasis model assessment for insulin resistance; HSCs, hematopoetic stem cells; IL, interleukin; IRS2, insulin receptor substrate 2; JAK2, Janus kinase 2; JVS, juvenile visceral steatosis; K18, keratin 18; K8, keratin 8; MBOAT7, membrane bound O-acyltransferase domain-containing 7; MCD, methionine- and choline-deficient; MDBs, Mallory-Denk bodies; MSG, monosodium glutamate; mTOR, mammalian target of rapamycin; NAFLD, non-alcoholic fatty liver disease; NAS, NAFLD activity score; NASH, non-alcoholic steatohepatitis; NEFA, non-esterified fatty acid; NFκB, nuclear factor NF-kappa-B; p53, tumor protein; PI3K, phosphatidylinositol 3-kinase; PKA, protein kinase A; PNPLA3, patatin-like phospholipase domain-containing protein 3; PPARs, peroxisome proliferator-activated receptors; PTEN, phosphatase and tensin homolog; PTEN-HEP-KO, hepatocyte-specific null mutation of PTEN in mice; SCD-1, stearoyl-CoA desaturase-1; SHP2, Src homology region 2 (SH2)-containing protein tyrosine phosphatase 2; Smad, TGF-β superfamily member; SREBP-1c, sterol regulatory element binding protein 1c; STAT, signal transducer and activation of transcription; STZ, streptozotocin; T2DM, type 2 diabetes mellitus; TG, triglyceride; TGF-β, transforming growth factor β; TM6SF2, transmembrane 6 superfamily member 2; TNFα, tumor necrosis factor α; US, United States; VLDL, very low-density lipoprotein; α-SMA, alpha-smooth muscle actin.
